# Avian Metapneumovirus in Thailand: Molecular Detection, Genetic Diversity, and Its Potential Threat to Poultry

**DOI:** 10.3390/v17070965

**Published:** 2025-07-09

**Authors:** Sudarat Wanarat, Manakorn Sukmak, Nantana Soda, Pimpakarn Suwan, Natchaya Satayaphongpan, Worata Klinsawat, Wilairat Chumsing, Chatnapa Janmeethat, Taweesak Songserm, Nuananong Sinwat, Sittinee Kulprasertsri, Pun Panomwan, Kriangkrai Witoonsatian

**Affiliations:** 1 Faculty of Veterinary Medicine, Kasetsart University, Kamphaeng Saen Campus, Nakhon Pathom 73140, Thailand; sudarat.wana@ku.th (S.W.);; 2 Department of Farm Resources and Production Medicine, Faculty of Veterinary Medicine, Kasetsart University, Kamphaeng Saen Campus, Nakhon Pathom 73140, Thailand; fvetmksu@ku.ac.th (M.S.);; 3 Kamphaeng Saen Veterinary Diagnostic Center, Faculty of Veterinary Medicine, Kasetsart University, Nakhon Pathom 73140, Thailand; 4 Conservation Ecology Program, School of Bioresources and Technology, King Mongkut’s University of Technology Thonburi, Bang Khun Thian Chai Thale Road, Tha Kham, Bang Khun Thian, Bangkok 10150, Thailand; 5 Department of Pathology, Faculty of Veterinary Medicine, Kasetsart University, Kamphaeng Saen Campus, Nakhon Pathom 73140, Thailand

**Keywords:** avian metapneumovirus, subtype-B, poultry, molecular detection, phylogenetic analysis, divergence time analysis, genetic diversity

## Abstract

Avian metapneumovirus subtype B (aMPV/B) is an economically significant pathogen in poultry, causing respiratory and reproductive disorders. In this study, 167 clinical samples were collected from commercial poultry farms across Thailand to investigate the prevalence, genetic diversity, and evolutionary dynamics of aMPV/B. Nested RT-PCR targeting the G gene revealed a positivity rate of 34.13% (57/167). Phylogenetic and Median-joining network analyses of sequenced amplicons identified two distinct Thai lineages: one genetically similar to vaccine strains and another of unknown origin. Divergence time analysis using a Bayesian framework estimated the time to the most recent common ancestor (tMRCA) of these lineages around 2006, with further sub-lineage diversification occurring around 2009 and 2016. These findings suggest that the circulating Thai aMPV/B strains likely stem from limited introduction events followed by local evolution. Lineage-specific amino acid substitutions within the G gene were identified, which may affect antigenic properties and immune recognition. This study highlights the molecular heterogeneity and ongoing diversification of aMPV/B in Thailand and underscores the need for sustained genomic surveillance and regionally tailored vaccination strategies.

## 1. Introduction

Avian metapneumovirus (aMPV), classified within the *Metapneumovirus* genus of the *Pneumoviridae* family, is a globally important pathogen responsible for upper respiratory disease in poultry species. First identified in turkeys in South Africa during the late 1970s [1], aMPV has since been divided into four major subtypes—A, B, C, and D—based on antigenic and genetic differences in the G gene, which encodes the viral attachment glycoprotein [2,3]. Subtypes A and B are the most prevalent in Europe, Asia, and Africa, whereas subtype C is primarily restricted to North America. Subtype D remains infrequently reported [4].

The G gene is notable for its high sequence variability, which plays a central role in immune evasion and host–virus interactions. This genetic plasticity complicates molecular diagnostics and vaccine development efforts [5]. Consequently, G gene-based analyses remain the cornerstone of aMPV subtyping and phylogenetic classification.

Among the subtypes, aMPV subtype B (aMPV/B) has garnered increasing attention due to its expanding host range and rising prevalence in chickens. Notably, the G gene is the most variable coding region of the aMPV genome and has been linked to changes in virulence, immunogenicity, and host adaptation [6,7].

In Southeast Asia, subtype B is the most frequently reported, with confirmed detections in both clinical cases and asymptomatic carriers. Investigations in Vietnam, China, and Malaysia have revealed multiple circulating field strains, some of which exhibit partial sequence identity with commercial vaccine strains [8,9,10]. These detections, which occurred in the absence of aMPV vaccination, raise important questions regarding natural viral evolution and environmental selection pressures.

Thailand, despite being a major poultry producer and exporter, has limited published data on the molecular epidemiology of aMPV. The first molecular detection of aMPV in the country was only reported in 2022, when subtype B was isolated from turkeys [11,12]. The paucity of surveillance data limits our understanding of the virus’s introduction routes, diversity, and evolutionary dynamics in the region.

Several factors may contribute to aMPV transmission and persistence, including cross-border poultry trade, migratory birds, and inconsistent implementation of biosecurity measures [13]. Similar findings have been reported in countries such as the United States and Brazil, where field strains with vaccine-like mutations have emerged in immunologically naïve flocks—suggesting in situ evolution rather than direct vaccine spillover [14].

Vaccination remains a key control strategy for aMPV. However, protection is not always correlated with antibody levels and may vary based on vaccine strain and administration protocols [15]. Furthermore, live vaccine strains have occasionally demonstrated environmental persistence and the capacity to regain pathogenicity under field conditions [16].

Global surveillance remains heterogeneous, with some countries conducting regular molecular monitoring and others—particularly in Southeast Asia—still relying on serological assays without genetic confirmation [17]. This inconsistency can obscure the true epidemiological picture and delay identification of emerging variants.

Recent advancements in real-time RT-PCR and high-throughput sequencing have improved genotyping accuracy and facilitated the detection of cryptic viral diversity. Nevertheless, in resource-limited settings, nested RT-PCR remains the most accessible and sensitive tool for detecting low viral loads in field samples [17]. In Thailand, the stakes are particularly high given the economic significance of poultry production and export. Despite reported seropositivity in several provinces, a comprehensive national genetic dataset of circulating aMPV strains is still lacking [18]. This gap poses both veterinary and trade-related risks, especially if divergent or pathogenic strains remain undetected.

Additionally, co-infection with other respiratory pathogens—such as *Mycoplasma gallisepticum*, infectious bronchitis virus (IBV), or avian influenza virus—can exacerbate clinical outcomes, complicate diagnosis, and interfere with vaccine efficacy. Therefore, integrated multi-pathogen surveillance is recommended to accurately assess the role of aMPV in respiratory disease complexes [19].

## 2. Materials and Methods

### 2.1. Sample Collection and Clinical Observation

A total of 167 biological specimens were collected from poultry flocks located across all major regions of Thailand, including the Northern, Northeastern, Central, Eastern, and Southern parts of the country, with several of these sample sources and methods previously documented in Wanarat (2024) [12]. This nationwide coverage was intentionally designed to capture the diverse ecological and management conditions of poultry production and to provide a comprehensive epidemiological overview of avian metapneumovirus (aMPV) occurrence under field conditions [11,17]. Sampling involved both symptomatic and asymptomatic birds in order to account for subclinical infections and gain an unbiased picture of viral circulation across different flock health statuses [8].

During farm visits, clinical evaluations were conducted by trained veterinary personnel. The most frequently observed clinical signs included nasal discharge, swelling of the infraorbital sinuses, conjunctivitis, respiratory distress, and notable reductions in egg production. These signs were used as guiding indicators for sample selection and for identifying potential involvement of aMPV in respiratory and reproductive disturbances [20,21].

To effectively target major tissues implicated in aMPV replication, a broad range of sample types was collected. Oropharyngeal swabs were obtained using sterile rayon-tipped applicators and transferred immediately into viral transport medium (VTM). Each oropharyngeal swab was collected from a different bird using aseptic technique and subsequently pooled (five swabs per pooled sample) in accordance with standard surveillance protocols to optimize detection while preventing cross-contamination [22,23]. Care was taken to ensure that each swab was collected separately prior to pooling to avoid cross-contamination. Swab samples were sourced from various commercial poultry farms nationwide, representing different production types and biosecurity levels.

For birds showing severe respiratory signs, necropsies were conducted and tracheal and lung tissues were harvested, as these organs are recognized as primary replication sites for aMPV [21]. In flocks with a history of decreased reproductive performance, postmortem samples of oviduct and ovarian tissues were also collected to investigate the potential role of aMPV in reproductive dysfunction. These reproductive organs are increasingly recognized as relevant targets, especially in laying hens experiencing reduced egg production or subfertility [24].

In addition to field-collected samples, a number of postmortem specimens were submitted by the Kamphaeng Saen Veterinary Diagnostic Center, part of the Faculty of Veterinary Medicine, Kasetsart University, located in Nakhon Pathom, Thailand. These samples were obtained through routine diagnostic services and contributed valuable insights into field cases of respiratory and reproductive disease in poultry.

Further supplemental specimens were collected opportunistically to enhance detection sensitivity and explore possible indirect markers of aMPV infection. These included allantoic fluid aspirated from embryonated eggs exhibiting shell thinning or abnormal development during hatchery operations, as well as infraorbital exudate and oviductal fluid collected from birds presenting with respiratory or reproductive abnormalities. Although these samples were not part of a controlled experimental setting, they were included to capture potential routes of viral exposure and transmission [9].

All specimens were handled under aseptic conditions and maintained within a cold chain throughout transportation. Samples intended for short-term testing were kept at −20 °C, while those requiring longer-term preservation were stored at −80 °C to maintain RNA integrity for subsequent nested RT-PCR and sequencing analyses [25,26].

Importantly, none of the birds sampled in this study had a documented history of aMPV vaccination, suggesting that the viral RNA detected most likely originated from natural exposure rather than vaccine-derived sources. This assumption, while not absolute, supports the interpretation of results by minimizing the likelihood of confounding signals and enabling clearer insights into the natural epidemiology and tissue tropism of the virus under field conditions [8,24].

This comprehensive and regionally inclusive sampling approach was developed to maximize the sensitivity of aMPV detection while also deepening our understanding of its clinical manifestations and tissue distribution in real-world poultry production systems.

### 2.2. RNA Extraction and Nested RT-PCR

Total RNA was extracted from pooled oropharyngeal swabs, respiratory and reproductive tissues, infraorbital discharge, oviductal fluid, and allantoic fluid using the Direct-zol™ RNA MiniPrep Kit (Zymo Research, Irvine, CA, USA), following the manufacturer’s instructions. Tissue samples were first homogenized in TRIzol^®^ reagent (Thermo Fisher Scientific, Waltham, MA, USA). All procedures were conducted under RNase-free conditions, and RNA was stored at −80 °C until further processing [22,27].

First-strand cDNA synthesis was performed using the RevertAid First Strand cDNA Synthesis Kit (Thermo Fisher Scientific, Waltham, MA, USA). Each 20 µL reaction mixture contained 4 µL of 5× reaction buffer, 2 µL of 10 mM dNTPs, 0.5 µL of RiboLock RNase inhibitor, 2 µL of random hexamer primers, and 1 µL of reverse transcriptase, with 10.5 µL of RNA template added on ice. The reaction was incubated at 42 °C for 60 min, followed by enzyme inactivation at 70 °C for 5 min [27].

To rule out concurrent infections with other common avian respiratory pathogens, all samples were screened using individual RT-PCR assays with specific primer sets for Newcastle disease virus (NDV), infectious bronchitis virus (IBV), *Mycoplasma gallisepticum*, and *Mycoplasma synoviae*. Only samples that were positive for aMPV/B and negative for all other tested pathogens were included in the molecular and phylogenetic analyses [4,28]. Inclusion criteria comprised birds exhibiting clinical signs consistent with aMPV infection, while exclusion criteria included recent vaccination history or detection of other respiratory pathogens.

Avian metapneumovirus (aMPV) detection was carried out via nested RT-PCR targeting the G gene, a region characterized by high variability that facilitates subtype differentiation [22,23]. The first (outer) PCR employed primers APV1.444G1+ and APV1/2.444G6−, amplifying a 444 bp fragment. Nested PCR reactions were subtype-specific: subtype A was detected using primers APV1.268G8+ and APV1.268.361G5− (268 bp product), while subtype B was identified using APV1.361G9+ and APV1.268.361G5− (361 bp product), following the protocol described by Rivera-Benítez et al. [22].

PCR was carried out in a 10 µL volume using Taq Phusion™ Hot Start II DNA Polymerase (Thermo Scientific, Waltham, MA, USA). The thermocycling profile consisted of an initial denaturation at 98 °C for 3 min; 40 cycles of denaturation at 98 °C for 30 s, annealing at 55 °C for 30 s, and extension at 72 °C for 30 s; followed by a final extension at 72 °C for 5 min [27].

Amplified PCR products were resolved on 1.5% agarose gels in 1× TAE buffer and visualized under UV light using an Alphadigidoc™ system (Alpha Innotech, San Leandro, CA, USA). Positive results were confirmed by the presence of bands corresponding to the expected sizes (268 bp or 361 bp). The commercial vaccine strain Hipraviar^®^ SHS (Laboratorios Hipra, Amer, Girona, Spain) served as a subtype B positive control.

### 2.3. Sequencing and Genetic Analysis

PCR products displaying clear bands at the expected sizes were selected for downstream sequencing. The selected amplicons were purified using the FavorPrep GEL/PCR Purification Kit (FAVORGEN Biotech, Taiwan, China), following the manufacturer’s protocol. Purified samples were then submitted for bidirectional Sanger sequencing through a commercial sequencing service (BTseq, U2Bio, Seoul, Republic of Korea) [22,24].

Raw chromatogram files were assembled and quality-checked using UGENE v52.0 (Unipro, Novosibirsk, Russia) [29]. Low-quality regions were trimmed, and high-confidence consensus sequences were generated. Multiple sequence alignment was performed using the MUSCLE algorithm integrated within UGENE [30]. Only sequences that fully covered the subtype-specific G gene fragment were retained for subsequent analysis.

Phylogenetic relationships were inferred using the Maximum Likelihood (ML) approach implemented in IQ-TREE v1.6.12 (Nguyen Lab, Vienna, Austria) [31,32]. ModelFinder was used to identify the best-fit nucleotide substitution model, with selection guided by the Bayesian Information Criterion (BIC). The robustness of the tree topology was evaluated through 1000 ultrafast bootstrap replicates and 1000 SH-aLRT tests [5].

The resulting phylogenetic tree was visualized and annotated using Interactive Tree of Life (iTOL) v6 (https://itol.embl.de, accessed on 2 January 2025) [33]. Reference sequences representing both vaccine-derived and field strains of aMPV subtype B were retrieved from GenBank (https://www.ncbi.nlm.nih.gov/genbank, accessed on 20 February 2024) [34] and included in the alignment for comparative analysis. Sequences generated in this study were submitted to GenBank with accession numbers and used for comparative phylogenetic analysis, providing transparency and ensuring traceability of the publicly available data [23,34].

### 2.4. Phylodynamic and Time-Calibrated Phylogenetic Analysis

Bayesian time-scaled phylogenetic analyses were conducted to estimate the evolutionary history and divergence times of avian metapneumovirus (aMPV) subtype B strains using BEAST v1.10.4 (BEAST Developers, University of Auckland, Auckland, New Zealand) [35]. A curated dataset comprising 192 partial sequences of the G gene—including Thai field isolates and reference strains from subtypes A to D—was aligned for downstream analyses.

The best-fitting nucleotide substitution model was determined using the W-IQ-TREE web server [31,32], with the General Time Reversible model incorporating gamma-distributed rate variation among sites (GTR + Γ4) and empirical base frequencies selected based on both Akaike and Bayesian Information Criteria (AIC/BIC). A strict molecular clock was applied, and its suitability was confirmed via marginal likelihood estimation using path-sampling and stepping-stone methods [36]. The clock rate prior followed a lognormal distribution with a mean in real space of 2.83 × 10^−3^ substitutions per site per year and a 95% highest posterior density (HPD) interval of 7.72 × 10^−4^ to 5.48 × 10^−3^, informed by previous studies [37].

The coalescent Bayesian Skygrid model [38] was selected as the tree prior based on superior model fit compared to the constant-size and Bayesian skyline models. Four independent Markov chain Monte Carlo (MCMC) runs were performed, each for 200 million generations with sampling every 10,000 steps. Log and tree files were combined using LogCombiner v1.10.4 (University of Auckland, Auckland, New Zealand) after discarding the first 20% of each run as burn-in. The maximum clade credibility (MCC) tree was summarized using TreeAnnotator v2.6.6 (University of Auckland, Auckland, New Zealand), with median node heights. Convergence and effective sampling of parameters were assessed using Tracer v1.7.2 (University of Auckland, Auckland, New Zealand), with all effective sample size (ESS) values exceeding 200 [39].

The final MCC tree was visualized and annotated using FigTree v1.4.4 (Andrew Rambaut, University of Edinburgh, Edinburgh, UK) [40], which allowed graphical presentation of node ages, posterior probabilities, and subtype groupings.

### 2.5. TCS Network Construction

To explore the genetic relationships and possible lineage diversification among Thai aMPV subtype B strains, a TCS (Templeton, Crandall, and Sing) network was constructed based on partial G gene sequences. Prior to analysis, all sequences were aligned using the MUSCLE algorithm [30]. Only distinct unique sequence types were included in the network to eliminate redundancy and highlight meaningful variation.

The TCS network was generated using PopART v1.7 (Population Analysis with Reticulate Trees, Leigh Lab, University of Otago, Dunedin, New Zealand), which implements the statistical parsimony approach for constructing haplotype networks based on genealogical relationships among closely related sequences [41,42]. This method connects haplotypes within a defined confidence limit of parsimony, providing a minimal mutational path among observed sequence types.

The dataset included both field-derived sequences from this study and reference sequences from two commercial vaccine strains—Hipraviar^®^ SHS and Nemovac^®^—allowing for assessment of potential divergence between vaccine and field lineages [24,37].

The final TCS network illustrated the genetic relationships and mutational connections among the unique sequence types. These findings complement the phylogenetic analysis and offer additional insights into the molecular epidemiology of aMPV subtype B in Thailand [23,37].

### 2.6. Amino Acid Sequence Comparison

To examine potential amino acid variation between field-derived and vaccine strains of aMPV subtype B, partial G gene nucleotide sequences were translated in silico into predicted amino acid sequences. Both the translation and alignment processes were carried out using UGENE v52.0 (Unipro, Russia) [29]. The translated sequences were then aligned using the MUSCLE algorithm to identify conserved regions as well as sites of amino acid variation [30].

The field isolate sequences obtained in this study were compared against reference protein sequences from two commercial vaccine strains—Hipraviar^®^ SHS and Nemovac^®^—retrieved from the NCBI GenBank database [34]. The residue differences were manually examined to highlight regions of potential divergence at the protein level [24].

This analysis aimed to determine whether the observed nucleotide substitutions led to synonymous or non-synonymous changes, which could have implications for antigenic properties or host immune recognition [4,8].

## 3. Results

### 3.1. Molecular Detection and Clinical Correlation of aMPV/B in Poultry

A total of 167 clinical samples were collected from poultry farms located across all five major geographic regions of Thailand—namely the North, Central, Northeast, East, and South—ensuring nationwide coverage and inclusion of diverse poultry production systems and species. The sampling strategy was intentionally designed to represent both symptomatic and asymptomatic birds, allowing for a comprehensive cross-sectional overview of avian metapneumovirus (aMPV) subtype B circulation throughout the country.

All samples were screened using a nested reverse transcription polymerase chain reaction (RT-PCR) assay targeting the G gene of aMPV. Out of the 167 samples, 57 (34.13%) tested positive for aMPV subtype B. No other subtypes (A, C, or D) were detected, consistent with current knowledge that subtype B is the predominant circulating strain in Southeast Asia, particularly within Thailand [11,12]. The remaining 110 samples (65.87%) were negative for aMPV.

**Figure 1:**
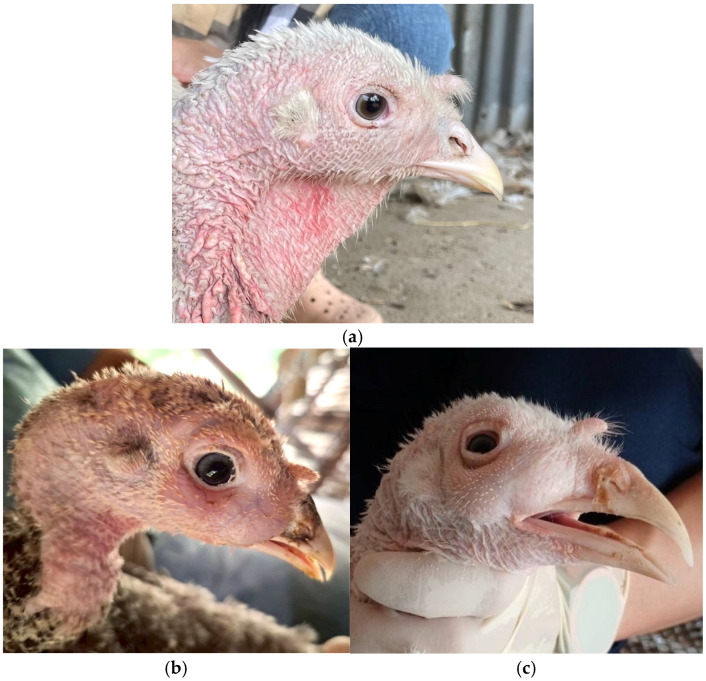
Clinical signs in turkeys associated with aMPV/B infection. (**a**) Clinically healthy turkey without respiratory symptoms, serving as a field control. (**b**) Turkey showing periocular edema and mild nasal discharge. (**c**) Turkey presenting with pronounced sinus swelling and mucoid nasal exudate.

Representative images illustrating clinical differences between uninfected and infected turkeys are shown in Figure 1a–c. Figure 1a depicts a healthy control turkey without clinical symptoms, while Figure 1b,c show naturally infected turkeys presenting hallmark signs of aMPV/B infection—namely, periocular edema, nasal discharge, and sinus swelling [43,44]. These images were obtained from field cases on commercial farms and not from experimental infection models. All birds were clinically assessed by veterinary diagnosticians during routine health monitoring visits.

Clinically, 143 birds (85.63% of total samples) showed signs consistent with upper respiratory tract infection. These included nasal discharge, infraorbital sinus swelling, foamy conjunctivitis, and periocular inflammation. Among the symptomatic birds, 54 (32.33%) were confirmed positive for aMPV/B by nested RT-PCR, whereas 89 birds (53.29%) tested negative despite exhibiting clinical signs. Notably, three PCR-positive birds (5.26%) did not present any clinical symptoms at the time of sampling. This indicates a partial overlap between clinical signs and molecular detection. Additional respiratory pathogens were identified in some flocks, including *Escherichia coli*, *Mycoplasma gallisepticum*, and Infectious Bronchitis Virus (IBV) [21,23,45]. These pathogens were detected through routine pathogen-specific RT-PCR screening conducted in parallel with aMPV/B testing.

Altogether, the incomplete overlap between clinical symptoms and molecular detection highlights the importance of integrating both diagnostic approaches. While molecular methods offer high specificity and are essential for accurate subtyping [22], clinical assessment remains a valuable tool for preliminary field screening and rapid response [46]. Employing a combined diagnostic strategy will strengthen future epidemiological surveillance and inform effective control programs targeting aMPV/B in poultry flocks across Thailand [25,37].

### 3.2. Phylogenetic Structure and Evolutionary Relationships

Phylogenetic analysis of partial G gene sequences from 57 aMPV/B-positive samples revealed the presence of two well-supported monophyletic clades, designated as Thailand Lineage I and Lineage II, which is consistent with previous findings from turkey samples in Thailand [12]. The maximum likelihood (ML) phylogenetic tree was constructed using IQ-TREE with 1000 ultrafast bootstrap replicates and SH-aLRT tests, yielding strong statistical support (bootstrap values >90%) across most major nodes [32,47].

A subset of Thai isolates—including PP117013, PP117015, and PP117019—formed a closely related, Thai-specific subclade within Lineage II. This clustering pattern points to localized viral evolution, possibly driven by clonal expansion within certain geographic areas or poultry systems [11,37]. Although genetically distinct from known reference strains, these isolates showed a loose phylogenetic association with older Asian sequences from Vietnam, China, and South Korea (e.g., MT432908, MT432909), suggesting a degree of shared ancestry or parallel regional diversification [5,48].

Notably, a few field isolates, such as PP117002, grouped in close proximity to sequences derived from commercial vaccines—specifically MZ574138 (Nemovac^®^) and MZ574139 (Hipraviar^®^). Although all Thai samples in this study were classified as field strains, the possibility that some viral sequences may reflect residual or environmental presence of vaccine strains—potentially introduced via undocumented vaccine use or movement of vaccinated birds—cannot be entirely ruled out. This consideration highlights the complexity of interpreting genetic similarity between field and vaccine strains in settings where vaccine usage may not be comprehensively recorded.

All Thai samples analyzed in this study were classified as field strains based on their genetic profiles. Some field isolates exhibited close phylogenetic relationships to vaccine-related sequences. In contrast, Thai field isolates were clearly distinct from European reference strains, such as Hungary/657/4 and VCO3/60616, which occupied basal or divergent positions in the phylogenetic tree [6]. Vaccine-associated sequences formed a separate and compact clade with no overlap observed with Thai field isolates [9].

**Figure 2:**
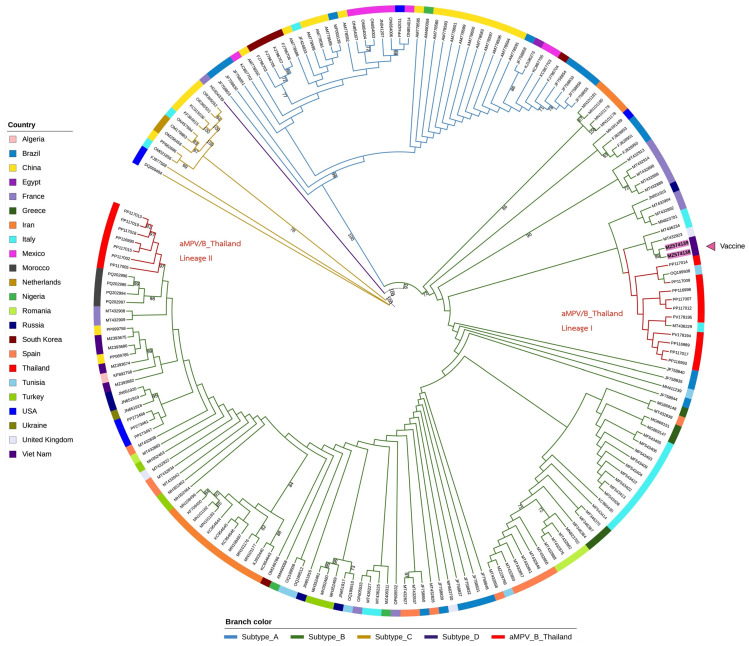
Maximum likelihood (ML) phylogenetic tree based on partial G gene sequences of avian metapneumovirus subtype B (aMPV/B), constructed using IQ-TREE. Thai field isolates formed two distinct clades, designated as Thailand Lineage I and Lineage II. Vaccine-derived reference strains (MZ574138, Nemovac*^®^* and MZ574139, Hipraviar*^®^*) formed a separate outgroup. The bootstrap values >70% are displayed at major nodes. Sequences from this study are labeled with GenBank accession numbers. The full-resolution tree is provided in Supplementary Figure S1.

These relationships are illustrated in Figure 2, which depicts the distinct clustering of Thai isolates, their divergence from vaccine strains, and the broader structure of global aMPV/B phylogeny.

Together, the phylogenetic data suggest that aMPV/B strains circulating in Thailand have undergone localized diversification, with evidence for the emergence of region-specific lineages and limited external introduction. Ongoing genomic surveillance, particularly through full-genome sequencing, will be essential for tracking evolutionary dynamics, assessing recombination risk, and informing the development of regionally effective vaccines [11,15].

All sequences used in the phylogenetic analysis were deposited in the NCBI GenBank database under the accession numbers PP116987–PP117019 and PV178194–PV178195. Detailed metadata, including host species, sampling locations, and lineage classification, are provided in Supplementary Table S1. This submission ensures traceability and enables further comparative genomic studies across regional and global datasets.

### 3.3. Divergence Time Analysis

**Figure 3:**
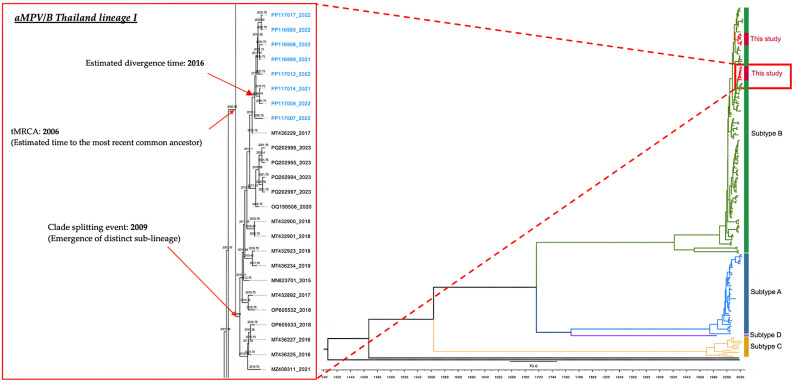
Time-scaled maximum clade credibility (MCC) tree of 193 avian metapneumovirus sequences, constructed using Bayesian analysis in BEAST. Subtypes A (blue), B (green), C (orange), and D (yellow) are shown. Thai Lineage I sequences from this study are marked in blue text. The tree suggests a most recent common ancestor (tMRCA) for the two Thai lineages around 2006, with divergence events occurring in 2009 and 2016. The complete tree is available in Supplementary Figure S2.

**Figure 4:**
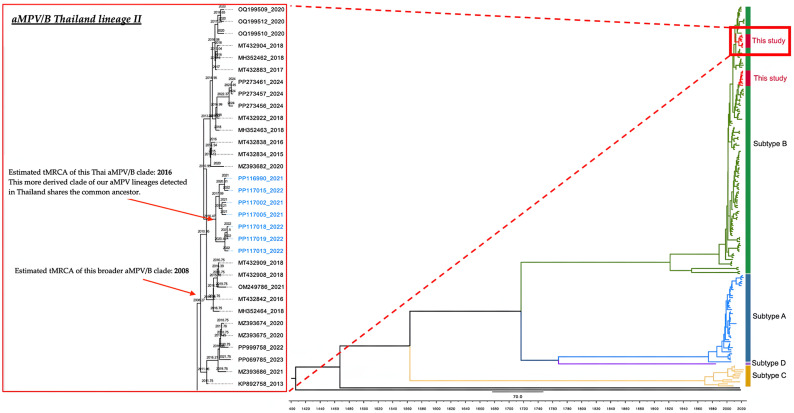
Expanded view of the derived Thai aMPV/B clade (Thailand Lineage II) from the MCC tree. Sequences are displayed in blue text. The lineage shares a tMRCA around 2008 and underwent further diversification circa 2016. The right panel situates this clade within the global subtype B context. Clock rate: 0.001919 substitutions/site/year (95% HPD: 0.0015301–0.002314). The full tree is provided in Supplementary Figure S2.

To gain deeper insights into the evolutionary dynamics of Thai avian metapneumovirus subtype B (aMPV/B) isolates, we carried out a time-scaled phylogenetic reconstruction using a dataset of 192 sequences. Sequences generated in the present study are highlighted in blue within the resulting maximum clade credibility (MCC) tree (Figure 3 and Figure 4). The estimated molecular clock rate was 0.001919 substitutions per site per year, with a 95% highest posterior density (HPD) interval between 0.0015301 and 0.002314, aligning well with previously reported evolutionary rates for aMPV [2,49].

Our analysis showed that Thai aMPV/B sequences grouped into two major clades, each representing distinct lineages within the broader subtype B population. The estimated time to the most recent common ancestor (tMRCA) for these two lineages was around 2006, a finding that echoes earlier observations of rapid diversification among aMPV/B lineages [50,51]. Notably, one of the clades underwent further divergence, giving rise to a separate sub-lineage by approximately 2009, pointing to independent evolutionary pathways within Thailand’s aMPV/B population.

Within the more derived clade, we identified a closely related subclade that includes the majority of contemporary Thai isolates. This cluster appears to have originated around 2016, Time-scaled phylogenetic analysis revealed that the broader lineage of Thai aMPV/B strains shared a common ancestor around 2008. A subsequent diversification event was estimated to have occurred in the mid-2010s [11,12].

The topology indicated that currently circulating strains are derived from a small number of ancestral nodes, consistent with limited introduction events and subsequent local diversification [52]. The patterns observed here emphasize the dynamic evolutionary nature of aMPV/B and underscore the critical need for continuous molecular surveillance to track emerging viral variants and inform effective disease control strategies.

### 3.4. TCS Network of aMPV/B Circulated in Thailand

Phylogenetic reconstruction and TCS network analysis revealed two genetically distinct lineages among Thai aMPV/B isolates: Thailand Lineage I and Thailand Lineage II. Although both fall within subtype B, the evolutionary trajectories and clustering patterns observed in each lineage were notably different, suggesting independent transmission histories [8,11,37].

Thailand Lineage I exhibited low genetic variability and a tightly clustered phylogenetic profile, indicative of a recent introduction or clonal expansion within a localized population [4]. In contrast, Thailand Lineage II displayed greater sequence diversity and a more dispersed distribution across both phylogenetic trees and TCS networks, pointing to a more complex evolutionary history and possibly longer-term circulation [6,11,12].

**Figure 5:**
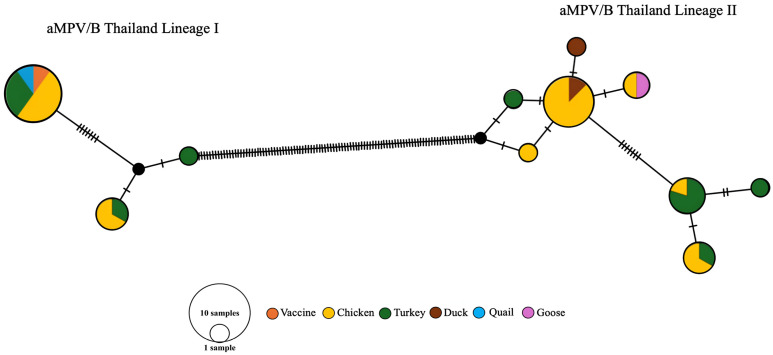
Median-joining TCS network of aMPV/B strains circulating in Thailand, constructed using PopART v1.7. Each circle represents a unique haplotype, and the size is proportional to the number of identical sequences. Colored segments indicate host species (chickens, turkeys, ducks, quails, geese). Hash marks on connecting lines denote nucleotide differences. Thailand Lineage I displays a star-like structure suggestive of recent expansion, while Lineage II forms a reticulated network indicative of prolonged circulation and genetic diversification.

To further explore these patterns, a TCS network was constructed using the statistical parsimony algorithm implemented in PopART v1.7 (Figure 5) [41,42]. The network structure reinforced the differences between lineages: Lineage I showed a star-like topology centered around a dominant sequence variant with minimal mutational steps to peripheral variants—consistent with rapid and recent spread. Meanwhile, Lineage II formed a more reticulated network with multiple sequence variants and intermediary nodes, suggestive of prolonged in-host evolution and possible recombination events [9,41].

These findings highlight the presence of lineage-specific molecular divergence among circulating aMPV/B strains in Thailand. Notably, both lineages were detected in various poultry species—including chickens, turkeys, ducks, and quails—indicating a potential for cross-species transmission and suggesting the existence of a shared viral reservoir [22,23].

Altogether, the co-circulation of distinct lineages across regions and species underscores the need for continuous molecular surveillance. Monitoring such lineage diversification over time will be essential to understanding transmission dynamics and guiding targeted control strategies in poultry populations [8,15].

### 3.5. Amino Acid Variation Between Lineages

Comparative analysis of the translated G gene sequences revealed consistent amino acid substitutions that clearly distinguish Thailand Lineage II from both Lineage I and the vaccine-derived reference strains. Specifically, three non-synonymous mutations were consistently identified in Lineage II isolates: Valine to Alanine at position 11 (Val→Ala), Glycine to Aspartic acid at position 40 (Gly→Asp), and Arginine to Histidine at position 74 (Arg→His). These substitutions were absent in all Lineage I samples and in vaccine strains MZ574138 and MZ574139 (representing Nemovac^®^ and Hipraviar^®^, respectively) [24,37].

A detailed summary of the amino acid substitutions identified among circulating Thai aMPV/B field strains is provided in Table 1. These substitutions were primarily located within the extracellular domain of the G protein. Sequence comparisons revealed consistent amino acid differences between field isolates and reference sequences [8,18].

Altogether, the presence of these consistent, lineage-specific substitutions adds further molecular support for the evolutionary divergence observed among aMPV/B strains in Thailand. Continued monitoring of these amino acid patterns may help uncover adaptive trends and guide the selection of strains for improved vaccine formulations and antigenic matching [3,8,15].

## 4. Discussion

This study provides a comprehensive molecular characterization of avian metapneumovirus subtype B (aMPV/B) currently circulating in poultry across Thailand. Using nested RT-PCR, targeted sequencing, and phylogenetic reconstruction, all positive samples were confirmed to belong exclusively to subtype B. This observation is consistent with previous reports from Southeast Asia, further supporting the notion that subtype B is the dominant—and likely endemic—lineage within this region [5,11,12,22]. The absence of other subtypes (A, C, and D) reinforces the importance of maintaining subtype B-specific diagnostics and control strategies [6,23].

Despite molecular detection, several aMPV/B-positive samples exhibited no observable clinical signs. This discrepancy suggests that viral presence does not always correlate with overt disease. One possibility is that birds were sampled during early stages of infection, prior to symptom onset [53]. Alternatively, subclinical infections may be more common than previously appreciated, particularly among partially immune birds or those with prior exposure to related strains. Yu et al. (2021) reported persistent viral shedding in asymptomatic birds, underscoring this hypothesis [7]. Mutations in the G gene, especially within its extracellular domain, may attenuate virulence while still allowing efficient replication and shedding [28]. Host- and environment-related variables—such as stress, coinfections, and age—likely contribute to this clinical variability [5]. These findings highlight the importance of integrating molecular diagnostics with field observations to capture the true epidemiological landscape of aMPV/B.

Phylogenetic analysis revealed that Thai aMPV/B isolates formed two principal lineages, here designated as Lineage I and Lineage II, each tracing back to a most recent common ancestor in the mid-2010s. Notably, a subset of isolates collected between 2021 and 2024 clustered tightly within a distinct clade supported by high posterior probability values, indicating recent divergence and suggesting localized viral circulation.

Historical field data provide important epidemiological context for these patterns. A turkey farm in northeastern Thailand, established in 2010, initially sourced poults from Ban Kham Koem, a community known for importing American Bronze turkeys in the early 2000s. Subsequent flock expansions in 2015 included birds from the Thap Kwang Livestock Research and Breeding Center, which maintains imported lines such as American Bronze and Beltsville Small White [12]. This two-tiered sourcing history is congruent with the bifurcation observed in the time-scaled phylogenetic tree, supporting the idea that geographically isolated founder populations contributed to the emergence of stable, distinct viral lineages.

Interestingly, sequences closely related to commercial vaccine strains (Nemovac^®^ and Hipraviar^®^) were detected on farms with no recorded history of aMPV vaccination. This raises critical questions regarding transmission dynamics, including potential environmental persistence, undocumented or off-label vaccine use, or introduction via migratory birds, as previously speculated in regional studies [4,9,37]. Nonetheless, all sequences were genetically distinct from vaccine strains, supporting a field origin. However, the close phylogenetic proximity of some isolates to vaccine-associated references suggests possible recombination or reversion events, warranting further investigation [8,24].

The two identified lineages exhibited specific non-synonymous substitutions at amino acid positions 11, 40, and 74 within the extracellular domain of the G protein, which may suggest molecular divergence. However, due to the limited number of available full-length vaccine strain sequences for direct comparison, it remains unclear whether these mutations represent definitive differences from the currently used vaccine strains. These mutations may have immunological implications by altering surface epitopes and facilitating immune evasion [3,18,54]. Lineage I showed high sequence similarity to existing vaccine strains, while Lineage II exhibited greater genetic divergence, especially within the hypervariable regions of the G gene. Such divergence may compromise neutralizing antibody recognition, as has been observed in Vietnam and Korea, where mismatches between circulating strains and vaccine antigens were associated with suboptimal immune protection and variable clinical outcomes [5,53].

The diagnostic approach employed in this study—a nested RT-PCR targeting the G gene—proved sensitive and specific for subtype B detection. However, its subtype specificity may limit detection of novel or divergent strains, underscoring the need for periodic primer re-evaluation as the virus evolves [2,27].

While the study provides valuable insights into the genetic structure of Thai aMPV/B, several limitations must be acknowledged. Certain avian species, particularly ducks, quails, and geese, were underrepresented, restricting conclusions about broader host susceptibility. Tracheal swabs yielded the highest positivity rates, but a more balanced and species-diverse. Sampling strategy could enhance future surveillance efforts. The lack of paired serological data also limits the ability to assess prior exposures or population-level immunity [26,46].

From a control perspective, the presence of vaccine-like sequences in non-vaccinated flocks and the emergence of lineage-specific antigenic mutations suggest that existing vaccines may not fully protect against evolving field strains. Updating vaccine compositions to better reflect circulating genetic variants—and improving documentation and regulation of vaccine use—should be prioritized [3,8,25]. Moreover, expanding sero-monitoring programs will be crucial for assessing vaccine performance and population immunity over time [26,45].

Future research should emphasize full-genome sequencing, broader host inclusion, and paired molecular and serological data. Such approaches are essential to better understand aMPV/B evolution, the potential for recombination, and the durability of protective immunity in diverse poultry systems [5,24,45].

## 5. Conclusions

This study provides a comprehensive molecular and evolutionary characterization of avian metapneumovirus subtype B (aMPV/B) in poultry across Thailand. All isolates were confirmed as subtype B and grouped into two genetically distinct lineages, likely resulting from separate introduction events. Divergence time estimates indicate that these lineages have circulated locally since at least 2006, with further diversification around 2009 and 2016. Some field isolates demonstrated high sequence similarity to vaccine strains, despite no reported history of vaccination. Lineage-specific non-synonymous mutations were observed within the G gene, particularly in regions associated with immune recognition.

While no vaccination was reported in the sampled flocks, the possibility of undocumented vaccine usage or the introduction of vaccinated birds into the population cannot be entirely excluded. Such scenarios, although sensitive, should be acknowledged as they may influence molecular interpretations and highlight the need for accurate flock health records. Continued genomic surveillance will be critical for tracking viral evolution and guiding effective control measures.

## Figures and Tables

**Table 1:** Amino acid alignment of partial G gene sequences of avian metapneumovirus subtype B (aMPV/B) vaccine strains and Thai field isolates. Highlighted positions (11, 40, and 74) indicate lineage-specific substitutions that differentiate Thailand Lineage II from Lineage I and vaccine strains. **Yellow shading** indicates amino acids conserved among vaccine strains and Thailand Lineage I. **Green shading** indicates substitutions uniquely found in Thailand Lineage II. All mutations are located in the extracellular domain of the G protein and may influence antigenicity. Amino acids are represented by the standard one-letter codes.

Group	Amino Acid Position	1	2	3	4	5	6	7	8	9	10	11	12	13	14	15	16	17	18	19	20	21	22	23	24	25	26	27	28	29	
Vaccine	Hipraviar^®^_MZ574138	V	I	S	I	C	I	S	V	E	Q	V	K	L	R	Q	C	V	D	T	Y	W	A	E	N	G	S	L	H	P	
	Nemovac^®^_MZ574139	V	I	S	I	C	I	S	V	E	Q	V	K	L	R	Q	C	V	D	T	Y	W	A	E	N	G	S	L	H	P	
aMPV/B Thailand Lineage I	KU07_PP116992	V	I	S	I	C	I	S	V	E	Q	V	K	L	R	Q	C	V	D	T	Y	W	A	E	N	G	S	L	H	P	
	KU10_PP116995	V	I	S	I	C	I	S	V	E	Q	V	K	L	R	Q	C	V	D	T	Y	W	A	E	N	G	S	L	H	P	
	KU11_PP116996	V	I	S	I	C	I	S	V	E	Q	V	K	L	R	Q	C	V	D	T	Y	W	A	E	N	G	S	L	H	P	
	KU18_PP117000	V	I	S	I	C	I	S	V	E	Q	V	K	L	R	Q	C	V	D	T	Y	W	A	E	N	G	S	L	H	P	
	KU28_PP117008	V	I	S	I	C	I	S	V	E	Q	V	K	L	R	Q	C	V	D	T	Y	W	A	E	N	G	S	L	H	P	
aMPV/B Thailand Lineage II	KU06_PP116991	V	I	S	I	C	I	S	V	E	Q	A	K	L	R	Q	C	V	D	T	Y	W	A	E	N	G	S	L	H	P	
	KU13_PP117013	V	I	S	I	C	I	S	V	E	Q	A	K	L	R	Q	C	V	D	T	Y	W	A	E	N	G	S	L	H	P	
	KU19_PP117001	V	I	S	I	C	I	S	V	E	Q	A	K	L	R	Q	C	V	D	T	Y	W	A	E	N	G	S	L	H	P	
	KU27_PP117019	V	I	S	I	C	I	S	V	E	Q	A	K	L	R	Q	C	V	D	T	Y	W	A	E	N	G	S	L	H	P	
	KU21_PP117003	V	I	S	I	C	I	S	V	E	Q	A	K	L	R	Q	C	V	D	T	Y	W	A	E	N	G	S	L	H	P	
**Group**	**Amino acid position**	**30**	**31**	**32**	**33**	**34**	**35**	**36**	**37**	**38**	**39**	**40**	**41**	**42**	**43**	**44**	**45**	**46**	**47**	**48**	**49**	**50**	**51**	**52**	**53**	**54**	**55**	**56**	**57**	**58**	
Vaccine	Hipraviar^®^_MZ574138	G	Q	S	T	E	N	T	S	T	R	G	K	T	T	T	K	D	P	R	R	L	Q	A	T	G	A	G	K	F	
	Nemovac^®^_MZ574139	G	Q	S	T	E	N	T	S	T	R	G	K	T	T	T	K	D	P	R	R	L	Q	A	T	G	A	G	K	F	
aMPV/B Thailand Lineage I	KU07_PP116992	G	Q	S	T	E	N	T	S	T	R	G	K	T	T	T	K	D	P	R	R	L	Q	A	T	G	A	G	K	F	
	KU10_PP116995	G	Q	S	T	E	N	T	S	T	R	G	K	T	T	T	K	D	P	R	R	L	Q	A	T	G	A	G	K	F	
	KU11_PP116996	G	Q	S	T	E	N	T	S	T	R	G	K	T	T	T	K	D	P	R	R	L	Q	A	T	G	A	G	K	F	
	KU18_PP117000	G	Q	S	T	E	N	T	S	T	R	G	K	T	T	T	K	D	P	R	R	L	Q	A	T	G	A	G	K	F	
	KU28_PP117008	G	Q	S	T	E	N	T	S	T	R	G	K	T	T	T	K	D	P	R	R	L	Q	A	T	G	A	G	K	F	
aMPV/B Thailand Lineage II	KU06_PP116991	G	Q	S	T	E	N	T	S	T	R	D	K	T	T	T	K	D	P	R	R	L	Q	A	T	G	A	G	K	F	
	KU13_PP117013	G	Q	S	T	E	N	T	S	T	R	D	K	T	T	T	K	D	P	R	R	L	Q	A	T	G	A	G	K	F	
	KU19_PP117001	G	Q	S	T	E	N	T	S	T	R	D	K	T	T	T	K	D	P	R	R	L	Q	A	T	G	A	G	K	F	
	KU27_PP117019	G	Q	S	T	E	N	T	S	T	R	D	K	T	T	T	K	D	P	R	R	L	Q	A	T	G	A	G	K	F	
	KU21_PP117003	G	Q	S	T	E	N	T	S	T	R	D	K	T	T	T	K	D	P	R	R	L	Q	A	T	G	A	G	K	F	
**Group**	**Amino acid position**	**59**	**60**	**61**	**62**	**63**	**64**	**65**	**66**	**67**	**68**	**69**	**70**	**71**	**72**	**73**	**74**	**75**	**76**	**77**	**78**	**79**	**80**	**81**	**82**	**83**	**84**	**85**	**86**	**87**	**88**
Vaccine	Hipraviar^®^_MZ574138	E	S	C	G	Y	V	Q	V	V	D	G	D	M	H	D	R	S	Y	A	V	L	G	G	V	D	C	L	G	L	L
	Nemovac^®^_MZ574139	E	S	C	G	Y	V	Q	V	V	D	G	D	M	H	D	R	S	Y	A	V	L	G	G	V	D	C	L	G	L	L
aMPV/B Thailand Lineage I	KU07_PP116992	E	S	C	G	Y	V	Q	V	V	D	G	D	M	H	D	R	S	Y	A	V	L	G	G	V	D	C	L	G	L	L
	KU10_PP116995	E	S	C	G	Y	V	Q	V	V	D	G	D	M	H	D	R	S	Y	A	V	L	G	G	V	D	C	L	G	L	L
	KU11_PP116996	E	S	C	G	Y	V	Q	V	V	D	G	D	M	H	D	R	S	Y	A	V	L	G	G	V	D	C	L	G	L	L
	KU18_PP117000	E	S	C	G	Y	V	Q	V	V	D	G	D	M	H	D	R	S	Y	A	V	L	G	G	V	D	C	L	G	L	L
	KU28_PP117008	E	S	C	G	Y	V	Q	V	V	D	G	D	M	H	D	R	S	Y	A	V	L	G	G	V	D	C	L	G	L	L
aMPV/B Thailand Lineage II	KU06_PP116991	E	S	C	G	Y	V	Q	V	V	D	G	D	M	H	D	H	S	Y	A	V	L	G	G	V	D	C	L	G	L	L
	KU13_PP117013	E	S	C	G	Y	V	Q	V	V	D	G	D	M	H	D	H	S	Y	A	V	L	G	G	V	D	C	L	G	L	L
	KU19_PP117001	E	S	C	G	Y	V	Q	V	V	D	G	D	M	H	D	H	S	Y	A	V	L	G	G	V	D	C	L	G	L	L
	KU27_PP117019	E	S	C	G	Y	V	Q	V	V	D	G	D	M	H	D	H	S	Y	A	V	L	G	G	V	D	C	L	G	L	L
	KU21_PP117003	E	S	C	G	Y	V	Q	V	V	D	G	D	M	H	D	H	S	Y	A	V	L	G	G	V	D	C	L	G	L	L

Note: Amino acids are represented by the standard one-letter codes—A: Alanine, C: Cysteine, D: Aspartic acid, E: Glutamic acid, F: Phenylalanine, G: Glycine, H: Histidine, I: Isoleucine, K: Lysine, L: Leucine, M: Methionine, N: Asparagine, P: Proline, Q: Glutamine, R: Arginine, S: Serine, T: Threonine, V: Valine, W: Tryptophan, Y: Tyrosine.

## Data Availability

The nucleotide sequences obtained in this study have been deposited in the NCBI GenBank database under accession numbers PV178194–PV178195 and PP116987–PP117017. These include trachea, oviduct, lung, allantoic fluid, swab, and infraorbital discharge samples collected from chickens, turkeys, ducks, quails, and geese.

## Supplementary Materials

The following supporting information can be downloaded at https://www.mdpi.com/article/10.3390/v17070965/s1. Supplementary Table S1: Metadata of avian metapneumovirus subtype B (aMPV/B) positive samples included in this study, including sample ID, host, location, date, and clade assignments based on G gene phylogeny. Supplementary Figure S1: Maximum likelihood phylogenetic tree based on partial G gene sequences of aMPV/B field and reference strains. Supplementary Figure S2: Maximum clade credibility (MCC) tree based on 193 avian metapneumovirus (aMPV) sequences reconstructed using Bayesian analysis. The tree displays global subtype relationships (subtypes A–D) and highlights the presence of two Thai-specific aMPV/B lineages. Sequences generated in this study are shown in blue text. The estimated molecular clock rate is 0.001919 substitutions/site/year (95% HPD: 0.0015301–0.002314).
