# Supplementary material for: Avian Metapneumovirus in Thailand: Molecular Detection, Genetic Diversity, and Its Potential Threat to Poultry
Source: Viruses. 2025 Jul 9;17(7):965. doi: 10.3390/v17070965 (PMC12300827; doi:10.3390/v17070965)
Supplement: Supplementary file 1 [file viruses-17-00965-s001.zip › Supplementary Material S1_Phylogenetic tree_revised.pdf]

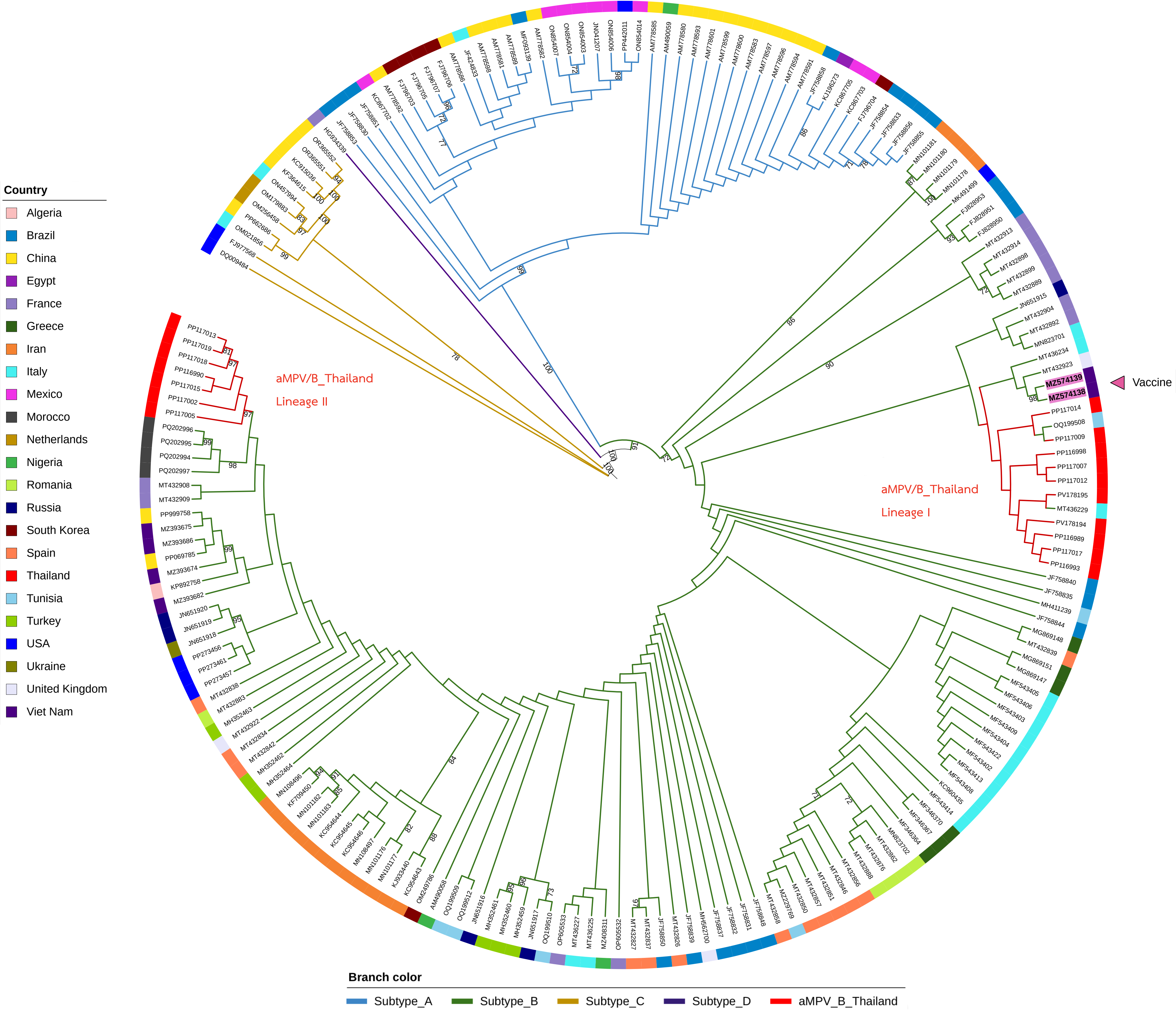

Country
Algeria
Brazil
China
Egypt
France
Greece
Iran
Italy
Mexico
Morocco
Netherlands
Nigeria
Romania
Russia
South Korea
Spain
Thailand
Tunisia
Turkey
USA
Ukraine
United Kingdom
Viet Nam
aMPV/B_Thailand
Lineage II
aMPV/B_Thailand
Lineage I
MZ574139
MZ574138
Vaccine
Branch color
Subtype_A
Subtype_B
Subtype_C
Subtype_D
aMPV_B_Thailand
