# Supplementary material for: Avian Metapneumovirus in Thailand: Molecular Detection, Genetic Diversity, and Its Potential Threat to Poultry
Source: Viruses. 2025 Jul 9;17(7):965. doi: 10.3390/v17070965 (PMC12300827; doi:10.3390/v17070965)
Supplement: Supplementary file 1 [file viruses-17-00965-s001.zip › Supplementary material S1_sample data.pdf]

Sample ID	Accession No.	Host species	Location (Province)	Collection date (YYYY-MM-DD)	G gene Clade (Thai lineage)	Remarks
KU01	PP116987	Duck	Nakhon Pathom	2022-01-06	Lineage II	
KU02	PP117014	Quail	Nakhon Pathom	2021-09-01	Lineage I	
KU03	PP116988	Chicken	Nakhon Pathom	2022-01-06	Lineage II	
KU04	PP116989	Chicken	Chonburi	2022-01-07	Lineage II	
KU05	PP116990	Turkey	Saraburi	2022-01-07	Lineage II	
KU06	PP116991	Chicken	Nakhon Nayok	2022-04-29	Lineage II	
KU07	PP116992	Chicken	Nakhon Pathom	2022-05-31	Lineage I	
KU08	PP116993	Chicken	Nakhon Pathom	2022-06-28	Lineage II	
KU09	PP116994	Chicken	Nakhon Pathom	2022-06-28	Lineage I	
KU10	PP116995	Chicken	Saraburi	2022-06-28	Lineage I	
KU11	PP116996	Chicken	Uttaradit	2022-07-15	Lineage II	
KU12	PP116997	Chicken	Phatthalung	2022-07-25	Lineage II	
KU13	PP117013	Chicken	Nakhon Ratchasima	2022-07-28	Lineage II	
KU14	PP116998	Duck	Nakhon Pathom	2022-08-19	Lineage II	
KU15	PP117015	Chicken	Chiang Rai	2022-08-24	Lineage I	
KU16	PP117018	Turkey	Saraburi	2022-09-08	Lineage II	
KU17	PP116999	Chicken	Phatthalung	2023-01-06	Lineage II	
KU18	PP117000	Chicken	Ratchaburi	2023-01-06	Lineage I	
KU19	PP117001	Chicken	Uttaradit	2023-01-06	Lineage I	
KU20	PP117002	Turkey	Kamphaeng Phet	2021-11-01	Lineage I	
KU21	PP117003	Turkey	Kamphaeng Phet	2021-11-01	Lineage II	
KU22	PP117004	Turkey	Kamphaeng Phet	2021-11-01	Lineage II	
KU23	PP117005	Turkey	Kamphaeng Phet	2021-11-16	Lineage II	
KU24	PP117016	Turkey	Ratchaburi	2022-03-23	Lineage I	
KU25	PP117006	Turkey	Ratchaburi	2022-03-23	Lineage I	
KU26	PP117007	Turkey	Ratchaburi	2022-03-31	Lineage II	
KU27	PP117019	Turkey	Ratchaburi	2023-03-08	Lineage I	
KU28	PP117008	Goose	Ratchaburi	2023-03-21	Lineage II	
KU29	PP117009	Chicken	Chiang Mai	2022-01-11	Lineage II	
KU30	PP117010	Chicken	Phra Nakhon Si Ayutthaya	2022-03-30	Lineage II	
KU31	PP117011	Chicken	Phra Nakhon Si Ayutthaya	2022-03-30	Lineage II	
KU32	PP117017	Chicken	Saraburi	2022-06-28	Lineage I	
KU33	PP117012	Chicken	Phatthalung	2022-07-25	Lineage I	
KU34	PV178194	Chicken	Chachoengsao	2021-07-09	Lineage I	
KU35	PV178195	Chicken	Nakhon Pathom	2021-06-26	Lineage II	
