# Supplementary figures and images for: Avian Metapneumovirus in Thailand: Molecular Detection, Genetic Diversity, and Its Potential Threat to Poultry
Source: Viruses. 2025 Jul 9;17(7):965. doi: 10.3390/v17070965 (PMC12300827; doi:10.3390/v17070965)

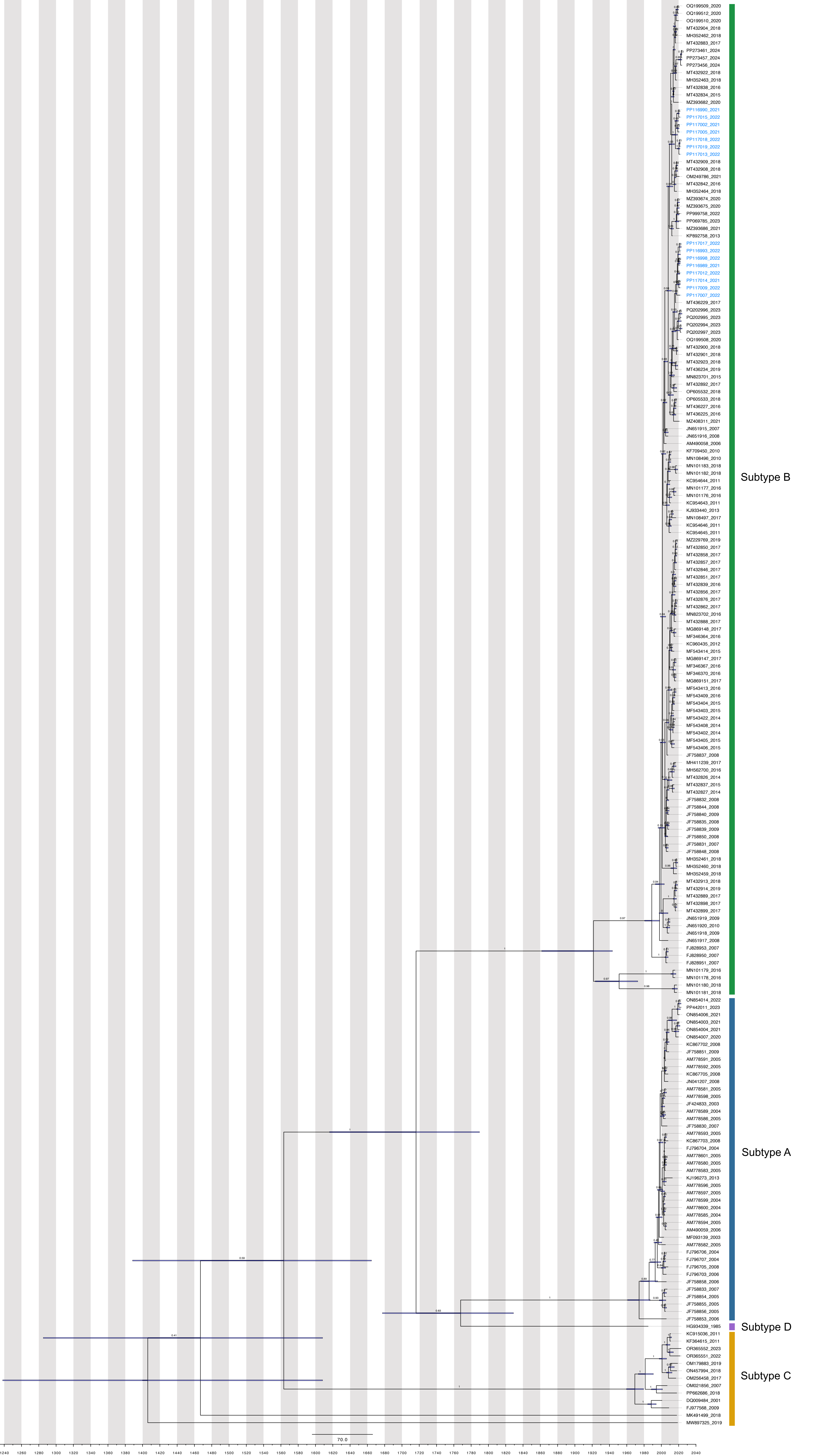

Supplement: Supplementary file 1 [file viruses-17-00965-s001.zip › Supplementary Material S2_Divergence time analysis.pdf]
